# Demolding Simulation of Propagation Phase Metasurfaces via Roll-to-Plate Nanoimprint

**DOI:** 10.3390/mi16121360

**Published:** 2025-11-29

**Authors:** Bowen Hu, Hao Chen, Dizhi Sun, Liangui Deng

**Affiliations:** 1 State Key Lab of Advanced Technology for Materials Synthesis and Processing, Wuhan University of Technology, Wuhan 430070, China; bowenhu@whut.edu.cn (B.H.); 359158@whut.edu.cn (H.C.); dizhis@whut.edu.cn (D.S.); 2 School of Information Engineering, Wuhan University of Technology, Wuhan 430070, China; 3 Suzhou Institute, Wuhan University, Suzhou 215028, China

**Keywords:** metasurface, roller nanoimprint, demold, finite element method

## Abstract

Propagation phase metasurfaces have excellent electromagnetic regulation and polarization-insensitive properties, while roll-to-plate nanoimprint lithography (R2P-NIL) is ideal for their large-scale low-cost fabrication. Existing demolding simulations for R2P-NIL are limited to 2D analysis, ignore elastomeric roller impacts, and cannot handle the discrete pillar/hole structures of such metasurfaces. This study establishes a 3D multiscale simulation model using a finite element method combining a macroscopic elastomeric roller deformation model and a microscopic demolding stress model with motion equation-based parameter transfer. Simulation results show macroscopically that zero elastomeric layer thickness minimizes stress, while stress rises and then stabilizes with increasing thickness; a moderately larger roller radius disperses stress; excessive pressure amplifies stress; a microscopically higher resist elastic modulus lowers stress; cylindrical structures have less stress than cuboids; and the limit aspect ratio peaks at a 100 nm line width. This work provides theoretical support for R2P-NIL parameter optimization and promotes the stable large-scale production of propagation phase metasurfaces.

## 1. Introduction

Metasurfaces are planar materials composed of arrays formed by artificial photonic structures, and they exhibit excellent electromagnetic regulation characteristics [1,2,3]. A propagation phase metasurface is a very important type of metasurface that possesses the characteristic of being polarization-insensitive. It has demonstrated precise phase manipulation capabilities in practical applications and can be combined with other phase control mechanisms (such as geometric phase) to achieve multi-degree-of-freedom modulation [4]. Depending on different configurations and shapes, propagation metasurfaces can achieve different functions, such as structural color patterns [5], metalenses [6,7], photonic integrated circuits [8], antireflection coating [9], etc.

In traditional metasurface fabrication technologies, electron beam lithography (EBL) can achieve nanoscale high-precision processing [10,11], but its high cost and low throughput severely limit the large-area, large-scale production of propagation phase metasurfaces. To address this issue, nanoimprint lithography (NIL) has emerged as an improved option. NIL is an important micro–nano-pattern fabrication technology that primarily achieves high-resolution pattern transfer via mechanical replication between a mold and a substrate. It boasts significant advantages that break the optical diffraction limit and feature a simple process, thus making it highly suitable for the low-cost and high-throughput fabrication of metasurfaces [12,13,14,15,16,17]. Compared with NIL, roller nanoimprint lithography (R-NIL) performs better in metasurface fabrication, as it not only has higher throughput but can also ensure pattern uniformity by controlling the pressure applied to the substrate [18,19,20,21].

The core process of R-NIL includes imprint resist filling, curing, and demolding, among which demolding directly determines the success of fabrication; even if the first two steps are perfectly completed, demolding failure will still lead to the bending or fracture of the nanostructure [22]. Furthermore, constrained by the material’s inherent refractive index, propagation phase metasurfaces inherently feature a high aspect ratio to achieve sufficient phase modulation capability, which in turn poses significant demolding challenges during the imprinting process. Simulation tools help us understand certain details, including the stress magnitude and distribution during the demolding process, which are difficult to obtain through experiments. Based on explicit dynamic finite element analysis, Liu et al. established simulation models for stress analysis of a rotating arm-based demolding system and roller-based demolding system, aiming to improve the demolding yield of polydimethylsiloxane (PDMS) microcolumns with an aspect ratio of 6 in automated systems [23]. More generally, the main task of demolding simulation is to optimize the fabrication process of NIL by studying the influence of factors such as adhesion and friction on demolding fidelity [24,25,26,27,28,29]. Guo et al. simulated adhesion and friction in the demolding cycle via finite element methods and proposed a method to optimize the demolding process using Ni-PTFE as the mold material to reduce surface energy and friction [30]. However, the aforementioned studies were limited to 2D analysis, lack analysis in the direction parallel to the roller, and are only applicable to wire grid-like structures, which are not suitable for comprehensive analysis of the discrete pillar or hole structures of propagation phase metasurfaces.

Additionally, to optimize the imprint resist filling step (a key part of the core process), elastic rollers (with elastomeric layers [31], air bladders [32], or multi-roller tensioned structures [33]) are often used in R2P-NIL. Yet, most of the research has not discussed the impact of such elastic rollers on the subsequent demolding process. Due to elastic rollers deforming under pressure and the introduction of extra macroscopic factors into the demolding, along with the higher computing power needed to simultaneously simulate both the nanoscale and meterscale with a single model, existing demolding simulation studies have not considered it. In conclusion, there is currently no simulation model for the demolding process of R2P NIL applied to propagation phase metasurfaces.

Thus, our work aims to address this gap by focusing on 3D demolding simulations for propagation phase metasurfaces fabricated via R2P NIL. Using the finite element method (FEM), an elastic composite roller deformation model and a propagation phase metasurface demolding stress simulation model were established, with parameter transfer between the two parts achieved through the equation of motion. This addresses the issue of excessive computational load caused by the inability of a single model to simultaneously handle both nanoscale and meterscale scenarios, enabling a demolding simulation that can simultaneously consider macroscopic factors (such as roller radius, applied pressure, and elastomeric layer thickness) and microscopic factors (such as pillar/hole structures and elastic modulus). This work provides theoretical references for the roll-to-plate (R2P) nanoimprint lithography of propagation phase metasurfaces and facilitates the optimization of imprint system structures and processing parameters.

## 2. Methods

**Figure 1:**
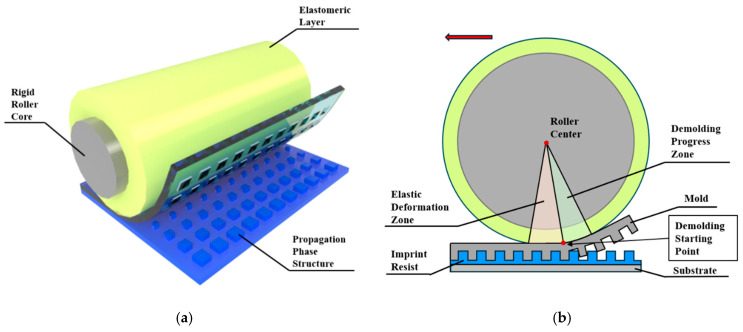
(**a**) Illustration of roller nanoimprint demolding; (**b**) 2D schematic of elastic roller demolding, the red arrow indicates the roller’s forward direction.

To accurately analyze the demolding stress distribution characteristics during the fabrication of propagation phase metasurfaces via R2P-NIL, we constructed a multiscale simulation framework based on the FEM. FEM numerical simulation analysis was conducted using the commercial finite element software COMSOL Multiphysics 6.2. Figure 1a illustrates how propagation phase metasurfaces are fabricated via R2P-NIL. The designed simulation model framework consists of two finite element models: one is the elastic roller deformation model for determining the deformation state of the elastic roller under pressure, and the other is the propagation phase metasurface demolding model for analyzing the stress distribution of the metasurface unit structure under macroscopic deformation parameters. The following sections gradually introduce the respective roles of the two models and how they are integrated.

**Figure 2:**
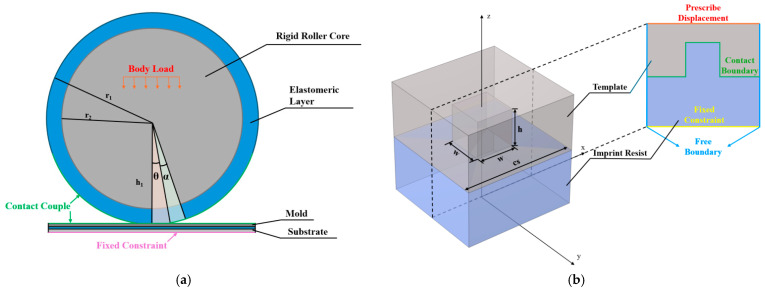
Multiscale simulation framework for demolding in elastic roller nanoimprint: (**a**) 2D macroscopic deformation model of elastic roller and its boundary conditions; (**b**) 3D microscopic demolding model and boundary conditions for propagation phase metasurface unit structures.

When a traditional rigid roller mold separates from the imprint resist, it initiates directly beneath the roller and detaches along the roller surface, following a full circular arc trajectory. In contrast, the elastic roller induces a localized elastic deformation zone at its bottom that alters the timing and position at which the mold separates from the imprint resist, as shown in Figure 1b. Within the elastic deformation zone, the mold undergoes only lateral movement with no significant separation taking place. Significant demolding commences when the demolding process reaches the end of the elastic deformation zone, which corresponds to the starting point of the demolding process zone; at this location, the mold formally initiates separation from the imprint resist. The entire separation process approximates circular motion around the roller so the motion trajectory can be determined by quantifying the dimensions of the elastic deformation zone shown as the red area (Figure 1b) and the demolding process zone shown as the green zone (Figure 1b). Here, the sizes of these two zones are characterized by angles *θ* and *α*, as illustrated in Figure 2a.

To determine the specific values of angles *θ* and *α* characterized by the elastic deformation zone and demolding process zone, we constructed an elastic roller deformation model. Figure 2a illustrates the macroscopic model and boundary conditions employed to determine the magnitudes of *θ* and *α*. Since the imprint roller exhibits axial translation symmetry, uniform axial pressure enables consistent cross-sectional deformation. This study adopted a simplified 2D model, which reduces computational load while maintaining high mesh precision. The roller is divided into a rigid core and an elastomeric layer to which a body load is applied to simulate mechanical pressure. This pressure is transmitted to the elastomeric layer inducing deformation therein. The bottom of the substrate was set with fixed constraints to prevent model movement caused by uneven forces.

Under a stable imprinting force, we first obtained the thickness change in the roller’s elastomeric layer Δ*h* through calculations in the simulation model. In the figure, the length of Δ*h* corresponds to the difference between the roller radius *r* and the height of the elastic deformation zone h_1_, i.e., Δ*h* = *r* − *h*_1_. Based on Δ*h*, the magnitudes of angles *θ* and *α* are further derived using trigonometric relationships, as described by the following formulas:

(1) $$\theta =\arccos\frac{r-∆h}{r}$$

(2) $$\alpha =\arccos\frac{r-∆h-h}{r}-\theta$$

where *h* represents the height of the structure. Inputting different parameters into the macroscopic model will change the magnitudes of *θ* and *α*, thereby affecting the start and end positions of mold separation. This further leads to changes in the mold deflection angle and influences the lateral force exerted on the microstructures. Such a phenomenon is absent in the demolding simulation analysis of traditional rigid roller models. The reason is that the lateral force induced by the elastic roller is affected by the macroscopic factors of imprinting and cannot be analyzed solely through the microscopic model. Therefore, introducing deformation parameters *θ* and *α* obtained from the macroscopic model into the microscopic model is intended to incorporate the influence of the elastic roller in the macroscopic model into the microscopic model.

In R-NIL, the factors influencing stress encompass three distinct force components, namely, adhesive force, frictional force, and lateral force. As illustrated in Figure 2b, a contact pair exists between the mold and the imprint resist, and this pair enables the definition of adhesive and frictional forces. Adhesion is realized through a cohesive zone model (CZM), where the cohesive energy parameter is automatically calculated using COMSOL based on the resist’s elastic modulus (800 MPa). Friction is defined by inputting a friction coefficient of 0.15, which is the intermediate value within the typical range (0.12–0.18) for common imprint resists (e.g., SU-8, PMMA) [18]. The lateral force by contrast originates from the rotational motion of the mold during demolding, and this motion induces deformation in the imprint resist structures. Therefore, obtaining the precise motion trajectory of the mold is critical.

Notably, due to the fixed constraints applied at the bottom, the origin of the coordinate system should be set here, as shown in Figure 2b. This setup directly determines the initial coordinates of the roller center. Specifically, the initial coordinates (*x_0_*, *z_0_*) of the roller center are given by

(3) $$\begin{array}{c} x_{0}=-r\sin\theta \\ z_{0}=r\cos\theta \end{array}$$

To simplify calculations, the total demolding time from initiation to completion is divided into discrete time intervals, where t represents the time variable within each interval, and *ω* denotes the angular velocity of the roller rotating around its center at any given moment. Within each interval, the mold undergoes a small incremental movement, and the von Mises stress distribution for that segment is computed. Through the parametric scanning of all time points, a complete demolding process can be reconstructed. The mold’s motion follows the equations outlined below, which describe its movement from the initial coordinates (*x, z*) to the new coordinates (*x*′, *z*′):

(4) $$\begin{array}{c} x^{\prime }=\left(x-x_{0}\right)\left(\cos\omega t-1\right)-\left(z-z_{0}\right)\sin\omega t-r\omega t\\ z^{\prime }=\left(x-x_{0}\right)\sin\omega t+\left(z-z_{0}\right)\left(\cos\omega t-1\right) \end{array}$$

where *θ* and *α* are the deformation parameters derived from the macroscopic model. When establishing boundary conditions, the bottom of the imprint resist is fixed with constraints, which means the mold’s motion is referenced to the imprint resist. Therefore, the position of the roller center must be precomputed using the roller radius and angle *θ*. By performing calculations with the aforementioned simulation model, we can obtain the dynamic stress distribution of the imprint resist at the microscale as well as the maximum stress value during the entire demolding process.

## 3. Results and Discussion

Building on the FEM simulation model of the R-NIL demolding process established in the previous sections, the present section presents in detail the results derived from FEM simulations and provides an in-depth discussion of these findings. The discussion centers on comparing the demolding performance of elastic rollers and rigid rollers, investigating the effects of macroscopic factors, namely, roller radius, elastomeric layer, thickness, and applied pressure, and analyzing the impacts of microscopic factors, including the elastic modulus of the imprint resist and columnar/hole structures. Finally, we investigated the influences of propagation phase unit structures with different shapes under different line widths and periods. Additionally, we presented the limit aspect ratios achievable with different line widths under a fixed period. In practical applications for models featuring symmetric structures, only half of the 3D model was utilized for calculations to reduce computational burden, and symmetric boundary conditions were applied at the cutting plane.

**Figure 3:**
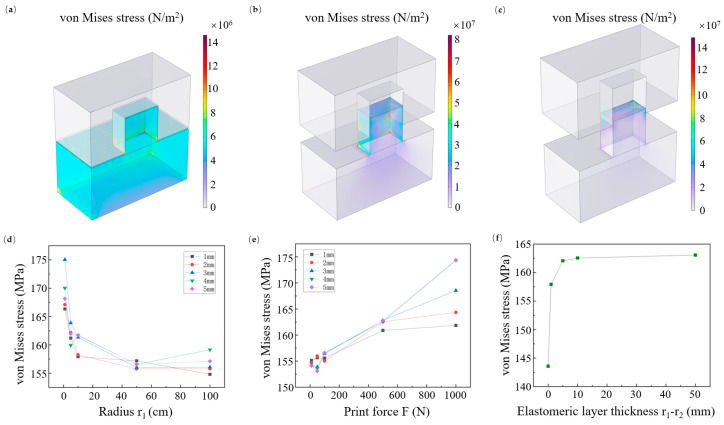
Typical stress distributions during demolding and effects of parameters on maximum stress in the imprint resist structure: (**a**) typical stress distribution at the initial separation between mold and imprint resist; (**b**) typical stress distribution when the mold moves upward along the sidewall; (**c**) typical stress distribution at the almost complete separation stage; (**d**) effect of roller radius on the maximum stress in the imprint resist structure under several elastic layer thicknesses; (**e**) effect of the imprint force applied to the roller on the maximum stress in the imprint resist structure; (**f**) effect of elastic layer thickness on the maximum stress of the imprint resist structure during the demolding process. For all simulations, parameters were fixed as follows unless otherwise specified: roller radius (*r*_1_) = 10 cm, imprinting force (*F*) = 300 N, aspect ratio = 1:1, and elastic modulus (*E*) = 800 MPa.

The changes in stress distribution as the demolding process progresses were investigated. As illustrated in Figure 3a, within the critical distance before adhesive failure occurs between the mold and the residual resist layer, stress is distributed across the imprint resist and begins to concentrate around nanostructures. After adhesive failure, stress localizes predominantly at the roots of the nanostructures, with peak values occurring at the bottom point aligned with the mold’s lateral separation direction (i.e., the bottom-right vertex in Figure 3b). As demolding progresses, stress increases as the mold is lifted. The rotational motion of the mold during roll demolding induces lateral extrusion forces at the resist–mold interface, which drives stress accumulation and explains the frequent bending of resist structures in R-NIL. During the final stage of mold separation, lateral forces impart a directional bias to the resist (rightward in Figure 3c), which exacerbates strain at the structure apex and amplifies stress concentrations at this location.

We further investigated macroscopic influencing factors that can be adjusted in elastic roller molds, including roller radius, applied pressure on the roller, and the thickness of the elastomeric layer in the elastic roller. Simulation results show that an excessively small roller radius hinders demolding (Figure 3d). Although increasing the roller radius helps reduce stress, a substantial increase in radius results in diminishing marginal returns, indicating that the roller size should be selected moderately. We also studied the effect of applied roller pressure on stress, a factor that conventional demolding simulation models cannot address. Excessive pressure increases structural stress, and this effect becomes more pronounced as the thickness of the elastomeric layer increases (Figure 3e). This phenomenon occurs because when the elastomeric layer is thin; stress increases significantly with an increase in its thickness (Figure 3f). Meanwhile, we found that the stress induced on the structure tends to stabilize once the thickness of the elastomeric layer exceeds a certain threshold. When the elastomeric layer thickness is zero (i.e., using a rigid roller), the stress induced on the structure decreases significantly (Figure 3f). This result indicates that although the elastomeric layer offers advantages for the filling and curing of imprint resist, it increases the difficulty of demolding. Therefore, in the design of an R2P NIL system, it is necessary to balance the trade-off between resist filling and demolding.

**Figure 4:**
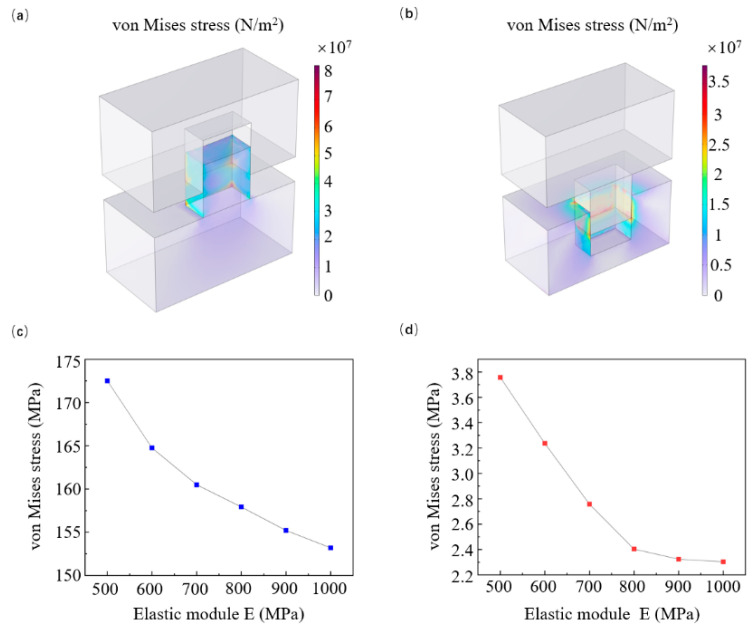
Demolding results of hole and pillar molds in R-NIL: (**a**) demolding stress distribution of hole mold; (**b**) demolding stress distribution of pillar mold; (**c**,**d**) variations in von Mises stress in pillar/hole structures under different elastic module. Blue line represents the stress change in the column structure, while red line represents the stress change in the hole structure. For all simulations, parameters are fixed as follows unless otherwise specified: roller radius (*r*_1_) = 10 cm, elastomeric layer thickness (*r_1_* − *r_2_*) = 1 mm, imprinting force (*F*) = 300 N, structure height (*h*) = 100 nm, and w = 100 nm.

When fabricating metasurfaces using R-NIL, we can choose between hole molds (Figure 4a) and pillar molds (Figure 4b). Although hole molds imprint the resist into pillar shapes, while pillar molds imprint it into hole shapes, the same structure can be obtained by selecting appropriate imprint resists. Simulation results clearly show that both types exhibit the same stress distribution variation pattern during demolding, but the maximum stress in pillar-shaped imprint resists (Figure 4c) is significantly higher than that in hole-shaped resists (Figure 4d). Therefore, using pillar molds to imprint hole structures is an effective approach to enhancing structural stability during demolding. However, this solution cannot be guaranteed to work in all cases. Our study found that increasing the elastic modulus of the imprint resist can reduce structural stress. Thus, adjusting the resist composition or directly using other resists with higher elastic moduli can enhance structural stability during demolding.

**Figure 5:**
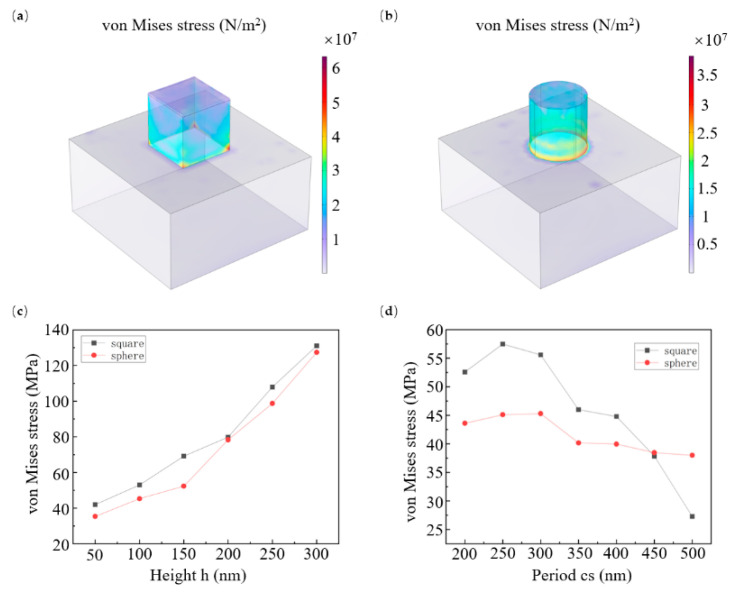
Stress contribution on cuboid (**a**) and column (**b**) imprint resist structure and residual layer; effects of height (**c**) and period (**d**) variations on the maximum stress magnitude in the cuboid and column imprint resist structures. For all simulations, parameters were fixed as follows unless otherwise specified: roller radius (*r*_1_) = 10 cm, elastomeric layer thickness (*r*_1_ − *r*_2_) = 1 mm, imprint force (*F*) = 300 N, structure height (*h*) = 100 nm, structure line width (*w*) = 100 nm, and imprint resist elastic module (*E*) = 800 MPa.

The line width of propagation phase metasurfaces unit structures is a core geometric parameter that directly regulates the equivalent refractive index and phase accumulation of the device by altering the duty cycle of the medium array. To explore how structural parameters affect mechanical stability during fabrication, we analyzed the stress variation in cuboid (Figure 5a) and cylindrical (Figure 5b) pillars under different structural heights and unit periods. As clearly shown in Figure 5c, with the line width kept constant (base side length for cuboids, diameter for cylinders), stress decreases as the structural height reduces. Additionally, the maximum stress in cuboid structures is slightly higher than that in cylindrical ones. This indicates more obvious stress concentration at the right-angle edges of cuboid bases compared to the curved surfaces of cylindrical bases. Beyond line width-related parameters, the period of unit structures also represents a critical design factor for propagation phase metasurfaces. As shown in Figure 5d, stress first increases and then decreases with the period rising at a fixed line width. Notably, the stress response of cylindrical pillars to period changes is significantly more muted than that of cuboid pillars, mainly because cylindrical surfaces disperse stress more evenly than the right-angle edges of cuboids.

**Figure 6:**
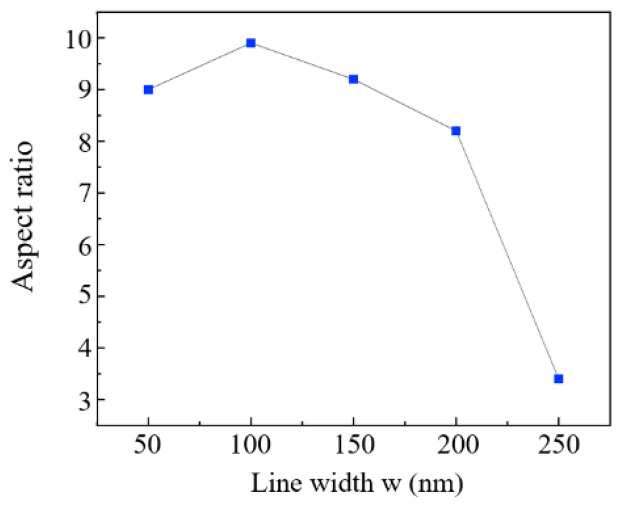
Illustrating the limit aspect ratios of propagation phase metasurface units with varying line widths. The results were obtained under a fixed process and geometric parameters as follows: period (*cs*) = 300 nm, roller radius (*r*_1_) = 10 cm, elastomeric layer thickness (*r*_1_ − *r*_2_) = 1 mm, and imprint force (*F*) = 100 N.

For propagation phase metasurfaces, their structures need sufficient height to meet the required phase modulation demands, as they are constrained by the material’s inherent refractive index. This inherent requirement directly leads to a high aspect ratio of metasurface units, which in turn presents significant challenges for large-scale fabrication using R2P NIL. We identified the limiting aspect ratio corresponding to different line widths of metasurface units with a fixed period of 300 nm via simulations. We aim to provide a processing size reference for the design of propagation phase metasurfaces. As illustrated in Figure 6, the limiting aspect ratio reaches a peak when the line width is 100 nm. For narrower line widths, severe stress concentration at the structure root means even small lateral forces induced by the rotation of the elastomeric roller during demolding can exceed the resist’s yield strength, leading to structural fracture. However, the sharp reduction in contact area reduces the total demolding force to a greater extent than the stress concentration elevates the fracture risk. Thus, narrower lines generally achieve a higher limiting aspect ratio compared to excessively wide ones such as over 200 nm. For overly wide lines such as 250 nm, the failure mode shifts from root fracture to global buckling instability. The structures bend rather than break, resulting in a sudden drop in the limit aspect ratio.

## 4. Conclusions

To address demolding failures like structural bending and fracture in fabricating propagation phase metasurfaces via R2P-NIL, we used FEM to build a multiscale simulation framework integrating a 2D macroscopic elastic roller deformation model and a 3D microscopic demolding stress model. Parameter transfer via motion equations solved the computational burden of single models handling both nanoscale and meterscale scenarios enabling the exploration of factors influencing the demolding fidelity comparison of elastomeric and rigid roller performance and derivation of limit aspect ratios of metasurface units under different line widths. Simulation results showed that for macroscopic factors, selecting an appropriate larger roller radius could reduce stress with diminishing returns, as well as that excessive pressure raised stress (more obvious with thicker elastomeric layers) and rigid rollers (no elastomeric layer) lowered stress for resists with good filling and curing. Microscopically, pillar molds for hole structures caused less stress, while selecting a higher elastic modulus resist could reduce stress, and cuboids had more stress than cylinders due to right-angle concentration. The limit aspect ratio peaked at 100 nm line width, narrower lines had a higher limit aspect ratio (greater demolding force reduction than fracture risk), and wider lines (>200 nm) saw a sharp limit aspect ratio drop from root fracture to buckling. This work fills two gaps overcoming traditional 2D simulation’s inability to reflect 3D metasurface features and ignoring the elastomeric roller’s demolding impact. Notably, the macroscopic elastomeric roller deformation model established in this study is applicable to all NIL techniques using elastic rollers for auxiliary demolding, the 3D microscopic demolding stress model is universally applicable to various NIL techniques, and the multiscale framework can be adapted to other roll-based demolding techniques such as R2R-NIL. The multiscale model solved the computational challenge of simulating nanoscale and meterscale simultaneously. Insights on roller selection structural design and parameter matching guide practical R2P-NIL, reducing demolding failures. This could provide theoretical guidance for the large-scale stable production of propagation phase metasurfaces, thereby promoting their industrial use in metalenses and other devices.

## Data Availability

The data that support the findings of this study are available from the corresponding author upon reasonable request.
